# Otolaryngology in the Time of Corona: Assessing Operative Impact and Risk During the COVID-19 Crisis

**DOI:** 10.1177/0194599820930214

**Published:** 2020-06-02

**Authors:** Hannah N. Kuhar, Ashley Heilingoetter, Maxwell Bergman, Noah Worobetz, Tendy Chiang, Laura Matrka

**Affiliations:** 1 Department of Otolaryngology–Head and Neck Surgery, Ohio State University Wexner Medical Center, Columbus, Ohio, USA; 2 Department of Pediatric Otolaryngology–Head and Neck Surgery, Nationwide Children’s Hospital, Columbus, Ohio, USA

**Keywords:** otolaryngology, COVID-19, coronavirus disease, SARS-CoV-2, preparatory response, airway management, intubation

## Abstract

**Objective:**

Limited research exists on the coronavirus disease 2019 (COVID-19) pandemic pertaining to otolaryngology–head and neck surgery (OHNS). The present study seeks to understand the response of OHNS workflows in the context of policy changes and to contribute to developing preparatory guidelines for perioperative management in OHNS.

**Study Design:**

Retrospective cohort study.

**Setting:**

Pediatric and general adult academic medical centers and a Comprehensive Cancer Center (CCC).

**Subjects and Methods:**

OHNS cases from March 18 to April 8, 2020—the 3 weeks immediately following the Ohio state-mandated suspension of all elective surgery on March 18, 2020—were compared with a 2019 control data set.

**Results:**

During this time, OHNS at the general adult and pediatric medical centers and CCC experienced 87.8%, 77.1%, and 32% decreases in surgical procedures as compared with 2019, respectively. Aerosol-generating procedures accounted for 86.8% of general adult cases, 92.4% of pediatric cases, and 62.0% of CCC cases. Preoperative COVID-19 testing occurred in 7.1% of general adult, 9% of pediatric, and 6.9% of CCC cases. The majority of procedures were tiers 3a and 3b per the Centers for Medicare & Medicaid Services. Aerosol-protective personal protective equipment (PPE) was worn in 28.6% of general adult, 90% of pediatric, and 15.5% of CCC cases.

**Conclusion:**

For OHNS, the majority of essential surgical cases remained high-risk aerosol-generating procedures. Preoperative COVID-19 testing and intraoperative PPE usage were initially inconsistent; systemwide guidelines were developed rapidly but lagged behind recommendations of the OHNS department and its academy. OHNS best practice standards are needed for preoperative COVID-19 status screening and PPE usage as we begin national reopening.

Coronavirus disease 2019 (COVID-19) is an acute infectious respiratory disease caused by the novel β-coronavirus SARS-CoV-2, or 2019 novel coronavirus (2019-nCoV). COVID-19 was recognized by the World Health Organization as a global pandemic on March 11, 2020.^1^ COVID-19 spreads primarily via respiratory tract droplets, secretions, and direct contact.^2^ Increasing evidence has demonstrated that procedures and examinations involving the upper aerodigestive tract pose a high risk for transmission.^3^ Particularly, the nose and nasopharynx are understood to be reservoirs for high concentrations of the SARS-CoV-2 virus.^4^ For this reason, the risk of transmission is high during maneuvers that involve the aerodigestive tract of patients with COVID-19. In these cases, the virus can spread via inhalation or mucosal contact with infected respiratory secretions.^5^

Otolaryngologists have been identified as a particularly vulnerable population among health care workers, as the majority of otolaryngologic procedures involve instrumentation of the upper aerodigestive tract.^6^ In the early stages of the pandemic, many health care workers, specifically non–primary care or consulting service providers such as otolaryngologists, were getting infected at higher rates as compared with other specialties.^7,8^ As nearly half the patients with COVID-19 present as afebrile and asymptomatic or with generalizable symptoms of nasal congestion, sore throat, and hyposmia, screening for clinical signs of COVID-19 infection is not effective to guide perioperative precautions.^7,9,10^ The possibility for occult positivity among children and adults who raise low clinical suspicion puts health care workers at risk of infection. For these reasons, otolaryngology examinations and aerosol-generating procedures (AGPs) are considered high risk for exposure from aerosol and droplet contamination by asymptomatic carriers of disease.^11^ Any procedure involving the mucosa of the aerodigestive tract is considered an AGP.^11,12^ Researchers posit that following manipulation of any of these areas, viral particles may be airborne for ≥3 hours.^13^

Recent safety guidelines on the recommended management of otolaryngologic cases suggest that examinations and procedures be limited to patients with clear indication and need, performed by the most experienced personnel available, and deferred if nonessential (ie, for a routine or lower-priority reason).^11^ A high-risk procedure is defined as surgery involving the nasal mucosa or contact with oral, pharyngeal, and pulmonary secretions.^11^ Researchers assert that the risk of transmission is highest during intubation, tracheostomy, and open airway procedures, which most often involve positive-pressure ventilation.^5^ Regarding surgical management of otolaryngologic cases, it is recommended that patient COVID-19 status be determined ahead of surgery, that high-risk operations be performed in negative-pressure operating rooms with appropriate personal protective equipment (PPE) worn by all staff, and that only essential staff be in the operating room for intubation and extubation.^11^ The American Academy of Otolaryngology–Head and Neck Surgery (AAO-HNS) released COVID-19-related resources, including patient screening algorithms and postexposure risk classifications.^5^ On March 18, 2020, the Centers for Medicare & Medicaid Services (CMS) released recommendations to delay all adult elective surgery and nonessential medical, surgical, and dental procedures during the COVID-19 response.^14^ CMS organized procedures into a series of tiers (1a-3b) meant to provide a framework for hospitals and clinicians to implement immediately during the COVID-19 response. The tier system takes into account patient risk factors; the availability of beds, staff, and PPE; and the urgency of the procedure.^14^

While guidelines on the perioperative management of otolaryngology–head and neck surgery (OHNS) cases are developing, there are several challenges to the implementation of such recommendations. One obstacle confronting otolaryngologists is the nationwide shortage of PPE necessary to perform surgical procedures.^15,16^ Additionally, the availability of timely COVID-19 testing has been limited due to regulatory processes and the time required to validate clinical tests, the initial lack of certified laboratories with polymerase chain reaction capabilities, and the shortage of chemicals and supplies.^17-19^ These limitations have restricted feasibility of consistent COVID-19 testing in the preoperative setting. Moreover, false-negative rates for these tests have been reported up to 21.4%.^20-22^ As national and local policies affecting the health care workforce change rapidly without consistent perioperative guidelines and adequate supplies, otolaryngologists are increasingly left to develop their own policies and practices to ensure surgeon and patient safety.

Limited research exists on the COVID-19 pandemic as it pertains to OHNS experiences, and urgent studies are required to characterize specialty response to the disease and streamline perioperative management. The purpose of the present study is to understand the impact of COVID-19 on perioperative workflows for OHNS at 2 tertiary academic medical centers in the context of national and state policy changes. The study focuses on the period since the Ohio state-mandated suspension of all elective surgery on March 18, 2020. This date was selected to capture the earliest phase of COVID-19 preparation in our state, prior to a peak in COVID-19 cases. This study examines institutional recommendations, department recommendations, society recommendations, and surgeon practices during this time. We seek to contribute to anticipatory efforts and preparatory guidelines for surgical planning and perioperative management in OHNS moving forward.

The objectives of the present study are 2-fold. First, we seek to examine the change in OHNS case volume and nature during the COVID-19 pandemic in the context of policy changes. We compare COVID-19 pandemic case numbers and types (March 18–April 8, 2020) directly with 2019 control data from the same date range, to understand the impact of the OHNS department response to policy changes. Second, we explore the spectrum of essential care that otolaryngologists are providing during COVID-19 in the adult and pediatric settings. We hypothesize that the majority of essential OHNS procedures performed remain high-risk (ie, AGPs) despite efforts to minimize surgical volume. We also examine the prevalence of perioperative COVID-19 testing and aerosol-protective PPE selection among otolaryngologists in response to the pandemic and national policy changes.

## Methods

### Study Design and Participants

This was a retrospective cohort study of all OHNS cases performed from March 18 through April 8, 2020, at a pediatric academic medical center and an adult academic medical center, inclusive of a Comprehensive Cancer Center (CCC). The study was approved by the Institutional Review Board of the Ohio State University Wexner Medical Center. Data were extracted from the electronic medical record through chart review.

### Data Collection

The following data points were extracted from electronic medical record chart review: COVID-19 history and symptoms, comorbid conditions (including immunosuppression, age >59 or <1 year, coronary artery disease or other heart disease, pulmonary disease), whether COVID-19 testing was performed, surgical procedure details (including inpatient/outpatient, CMS tier, primary *International Classification of Disease, Tenth Revision* code, and *Current Procedural Terminology* code), case airway management (intubation, laryngeal mask airway, bag mask, spontaneous or jet ventilation, ventilation through tracheostomy), and PPE utilized. A case was determined to be mucosal or an AGP if it involved the mucosa of the head and neck, specifically within the nose, sinuses, nasopharynx, oral cavity, oropharynx, larynx, trachea, mastoid or middle ear, and esophagus.^11^ Rationale for including the esophagus is that instrumentation of the upper airway is required to access.

Additional data points collected included case volumes and types from March 18 through April 8, 2019, as a reference point for direct comparison with data for March 18 through April 8, 2020. Additionally, all scheduled OHNS cases were captured that were deemed elective and subsequently canceled from March 18 and April 8, 2020. A timeline of events from March through April 2020 was designed to capture policy changes related to the COVID-19 response at national, state, local institutional, and departmental levels. Data on use of aerosol-protective PPE were collected from surgeons directly when not noted in the electronic medical record. On April 2, 2020, a standardized template was instituted to capture data regarding airway management and COVID-19 testing and status, as well as PPE usage by surgeons, staff, and anesthesia. This template was included by attending and resident surgeons at the end of brief operative notes. PPE information was collected from these templates when available.

### Statistical Analyses

Descriptive statistical analyses were performed. Categorical variables were described as frequency rates and percentages. All statistical analyses were performed with Microsoft Excel.

Analyses included cases from a pediatric academic medical center and an adult academic medical center, inclusive of a CCC. Data were collected for March 18 to April 8, 2020, which includes the 3 weeks immediately following the state-mandated suspension of all nonelective procedures in Ohio. Data were also collected for March 18 to April 8, 2019, to compare case volume and procedure type between 2019 and 2020 for the same date range. Comprehensive data were collected on each surgical case, including types of procedures performed, as many cases comprised ≥2 procedures.

## Results

### Case Data

**Figure 1.**
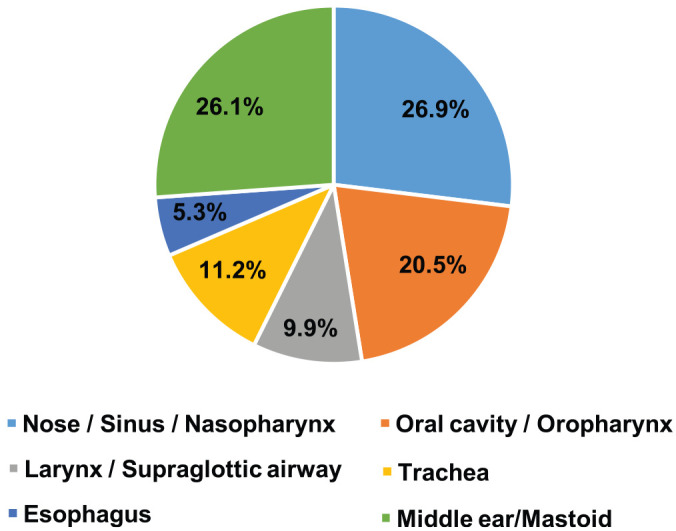
Aerosol-generating procedures at all 3 sites by anatomic location: March 18 to April 8, 2020.

From March 18 to April 8, 2020, there were 14 general adult cases (38 procedures), 142 pediatric cases (225 procedures), and 58 CCC adult cases (221 procedures). Canceled cases during this time frame included 258 general adult, 418 pediatric, and 46 CCC adult. Of the general adult procedures, 86.8% were AGPs; of pediatric procedures, 92.4%; of CCC adult procedures, 62.0% (**Table 1**). Anatomic locations of AGPs performed across all 3 sites included 26.9% for the nose, sinus, and nasopharynx; 26.1% for the middle ear and mastoid; 20.5% for the oral cavity and oropharynx; 11.2% for the trachea; 9.9% for the larynx and supraglottic airway; and 5.3% for the esophagus (**Figure 1**).

**Table 1.** Descriptive Information of Cases at All 3 Sites: March 18–April 8, 2020.^a^

	Adult academic medical center	Pediatric academic medical center	Cancer care center
Case data			
Cases	14	142	58
Canceled cases	258	418	46
Total procedures	38	225	221
Aerosol-generating procedures	86.8 (33)	92.4 (208)	62.0 (137)
Preoperative COVID-19 testing	7.1 (1)	9 (13)	6.9 (4)
COVID-19 positive	0	0	0
CMS tier 3a procedures	71.4 (10)	71.1 (101)	81 (47)
CMS tier 3b procedures	7.1 (1)	28.2 (40)	15.5 (9)
CMS tier 2 procedures	7.1 (1)	0.7 (1)	1.7 (1)
CMS tier 1 procedures	14.3 (2)	0	1.7 (1)
Patient data			
Mean age, y	46.4	3.4	59.4
Female	35.7 (5)	52.5 (71)	50 (27)
No comorbidities	50 (7)	69.6 (94)	13.0 (7)
Immunosuppressed	28.6 ^b^ (4)	0.7 (1)	87.0 ^b^ (47)
Age <1 or >59 y	35.7 (5)	34.1 (46)	57.4 (31)
Heart disease	42.9 (6)	4.4 (6)	40.7 (22)
Pulmonary disease	14.3 (2)	8.9 (12)	20.3 (11)
Other comorbidities	7.1 (1)	5.9 (8)	13.0 (7)
Airway management data			
Intubation	92.9 (13)	47.2 (67)	96.6 (56)
Laryngeal mask airway	0	0	0
Bag mask ventilation	0	35.2 (50)	0
Spontaneous ventilation	0	16.2 (23)	0
Jet ventilation	7.1 (1)	0	0
Ventilation via tracheostomy	0	1.4 (2)	3.4 (2)

Abbreviation: CMS, Centers for Medicare & Medicaid Services.

a Values are presented as No. or % (No.) unless noted otherwise.

b Immunosuppressed secondary to malignancy.

Preoperative COVID-19 testing was performed in 7.1% of general adult cases, 9% of pediatric cases, and 6.9% of CCC cases. No tested patients were COVID-19 positive at any of the 3 sites. General adult procedures included 71.4% CMS tier 3a, 7.1% CMS tier 3b, 7.1% CMS tier 2, and 14.3% CMS tier 1. Pediatric procedures included 71.1% CMS tier 3a, 28.2% CMS tier 3b, 0.7% CMS tier 2, and no CMS tier 1. Procedures performed at the CCC included 81% CMS tier 3a, 15.5% CMS tier 3b, 1.7% CMS tier 2, and 1.7% CMS tier 1. All data are summarized in **Table 1**.

### Patient Demographics

Of the general adult patients, 35.7% were female, and their mean age was 46.4 years. Of general adult patients included in this study, 50% had no comorbidities; 42.9% had heart disease; 35.7% were ≥59 years old; 28.6% were immunocompromised secondary to malignancy; 14.3% had pulmonary disease; and 7.1% had other comorbidities.

Among pediatric patients, 52.5% were female, and their mean age was 3.4 years. Of the pediatric patients included in this study, 69.6% had no comorbidities; 34.1% were <1 year old; 8.9% had pulmonary disease; 5.9% had other comorbidities; 4.4% had heart disease; and 0.7% were immunocompromised.

At the CCC, the patient population was 50% female and averaged 59.4 years of age. Of CCC patients included in this study, 87.0% were immunocompromised secondary to malignancy; 57.4% were ≥59 years old; 40.7% had heart disease; 20.3% had pulmonary disease; 13.0% had other comorbidities; and 13.0% had no comorbidities.

All data are summarized in **Table 1**.

### Airway Management and PPE

Of the general adult patients, 92.9% were intubated for procedures; 7.1% underwent jet ventilation; and no patients underwent ventilation via tracheostomy or bag mask ventilation as the sole form of perioperative ventilation. Of the pediatric patients, 47.2% were intubated for procedures; 35.2% underwent bag mask ventilation; 16.2% underwent spontaneous ventilation; and 1.4% were ventilated via tracheostomy. At the CCC, 96.6% of patients were intubated for procedures; 3.4% were ventilated via tracheostomy; and no patients underwent bag mask ventilation, spontaneous ventilation, or jet ventilation. Data on bag mask ventilation on emergence or during transportation were not available. Laryngeal mask airway was not used at any of the 3 sites. All data are summarized in **Table 1**.

In this study, “aerosol-protective PPE” means N95 mask and full eye protection, as opposed to regular surgical mask and loupes, for instance. PPE data were collected from direct questioning of surgeons at all 3 sites or from standardized templates in brief operative notes. At the general adult medical center, surgeon response rates were 100%. At the pediatric medical center, there was an overall surgeon response rate of 70.4% (100 of 142) regarding PPE. At the CCC, surgeon response rates were 100%. PPE was worn by surgeons in 28.6% of general adult cases (33.3% of AGP cases), 90% of pediatric cases (89.4% of AGP cases), and 15.5% of CCC cases (15.8% of AGP cases). At the general adult medical center, PPE usage increased from 25% during week 1 (March 18-24) to 33.3% during week 2 (March 25-31); no cases were performed at the general adult medical center during week 3. PPE was used in 94.5% of pediatric cases (63.2% response rate) during week 1, 88.8% (94.7% response rate) during week 2, and 66.6% (52.9% response rate) during week 3. At the CCC, PPE usage increased from 14.8% during week 1 (March 18-24) to 23.1% during week 3 (April 1-8).

### 2019 Data

**Figure 2.**
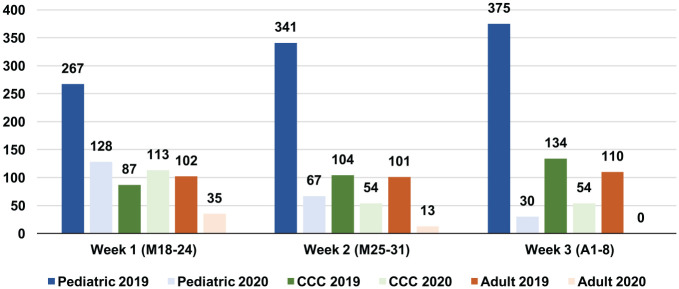
Number of overall procedures performed by week for all 3 sites: March 18 to April 8, 2019 vs 2020. CCC, Comprehensive Cancer Center.

Procedure volume and type were collected for March 18 to April 8, 2019, across all 3 sites. During this period, 313 general adult procedures were performed, of which 307 were AGPs (98.1%). A total of 983 pediatric procedures were performed, of which 925 were AGPs (94.1%), and 325 CCC adult procedures were completed, of which 236 were AGPs (72.6%). Comparison of 2019 and 2020 surgical volume by week across all 3 sites is summarized in **Figure 2**. General adult, pediatric, and CCC medical centers experienced 87.8%, 77.1%, and 32% decreases in surgical volume as compared with 2019, respectively. From March 18 to April 8, 2020, general adult, pediatric, and CCC medical centers canceled 258, 418, and 46 cases, respectively (**Table 1**).

## Discussion

Proper preparation and health system response in the setting of a pandemic involve the implementation of social precautions, medical resource conservation and reallocation, and development of standardized best practices responsive to the situation at hand. Also of importance is the protection of all members of the perioperative ecosystem from potential infection. As a result, many states have mandated the suspension of elective procedures for staff safety as well as resource preservation. Despite dramatic de-escalation of overall surgical volume, we identified that otolaryngologists remain at high risk when providing essential care during the COVID-19 pandemic due to the overwhelming proportion of AGPs forming their case load. The goal of the present study is to describe the responses of a health care system and OHNS departments to inform future preparedness efforts.

**Figure 3.**
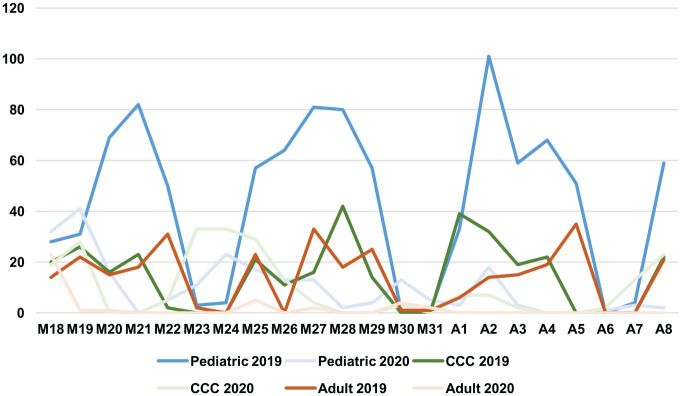
Number of overall procedures performed by day for all 3 sites: March 18 to April 8, 2019 vs 2020. CCC, Comprehensive Cancer Center.

**Figure 4.**
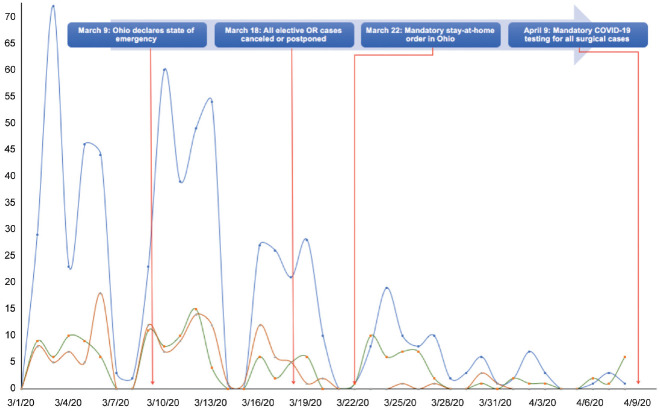
Number of otolaryngology surgical procedures at all 3 sites by date with associated timeline of major state- and hospital-level recommendations.

During the COVID-19 pandemic, OHNS departments proactively responded to institutional, national, and state mandates by adjusting operative case volumes and types. In the 3 weeks immediately following the Ohio state-mandated suspension of all nonelective surgery on March 18, 2020, general adult, pediatric, and CCC medical centers experienced 87.8%, 77.1%, and 32% decreases in surgical volume as compared with 2019, respectively. As seen in **Figure 3**, surgical case volume decreased significantly across all medical centers following the March 18 mandate. The decreasing number of OHNS surgical procedures performed across all medical centers was associated with major state- and hospital-level recommendations (**Figure 4**). Over 700 cases were canceled in a 3-week period (**Table 1**). The greatest impact on case volume occurred at the general adult medical center, where the majority of cases are elective and outpatient procedures. Guidelines evolved most rapidly during the third week of the study, during which a brief hiatus in surgery occurred at the general adult medical center while policies were more firmly characterized. Case volume at the CCC decreased, though not as significantly as that at the pediatric and adult medical centers, likely due to the comparatively more urgent nature of the oncologic cases at the CCC during this time. Of patients who underwent surgery at the CCC during the COVID-19 response, 87.0% had an established cancer diagnosis. The nationally mandated CMS tier criteria also affected the types of surgical cases that took place across all 3 centers during this time. The majority of procedures performed were CMS tier 3a: 71.4% of general adult cases, 71.1% of pediatric cases, and 81% of CCC cases. All CMS tier 2 and 1 cases (n = 5) at the CCC and general adult medical center during this period occurred between March 18 and 19, 2020 (**Table 1**). State and national mandates affected the volume and nature of cases encountered across all 3 sites. Cases performed from March 18 to April 8, 2020, were fewer in number but greater in urgency.

AGPs have been established in the limited existing COVID-19 literature to be higher risk for viral transmission due to the potential for viral particles to become aerosolized during mucosal procedures that involve the upper aerodigestive tract.^3,4^ In this study, we defined AGP as any procedure involving mucosal surfaces of the nose, sinuses, nasopharynx, oral cavity, oropharynx, larynx, trachea, esophagus, and middle ear/mastoid. During March 18 to April 8, 2019, AGPs made up 98.1% of all general adult procedures, 94.1% of all pediatric procedures, and 72.6% of all CCC procedures. Although the number of overall OHNS procedures decreased during this time, the proportion of AGPs did not change significantly across these 3 medical centers. Of the general adult, pediatric, and CCC procedures performed during the COVID-19 pandemic and response, 86.8%, 92.4%, and 62.0% were AGPs, respectively (**Table 1**).

Despite widespread recognition that AGPs are particularly high-risk procedures for COVID-19 transmission, the present study demonstrates that AGPs remained essential and often unavoidable in the field of OHNS during this time. For 3 medical centers in the immediate COVID-19 response period, AGPs represented a substantial proportion of otolaryngologic surgical cases.

While AGPs continued, changes in perioperative management occurred in the immediate COVID-19 response period of March 18 to April 8, 2020. Preoperative COVID-19 testing took place in 7.1% (n = 1) of general adult cases, 9% (n = 13) of pediatric cases, and 6.9% (n = 4) of CCC cases (**Table 1**). The limited amount of COVID-19 testing performed during this time reflects the known nationwide shortage of timely and readily available testing.^15-19^ Such limitations restricted the ability to efficiently integrate consistent COVID-19 testing into the preoperative setting of 3 academic medical centers in the first 3 weeks of the pandemic response. This encouraged the necessary development of a perioperative risk management infrastructure. While OHNS society recommendations call for determination of patient COVID-19 status prior to surgery, the reality of operationalizing such a requirement is extremely difficult in the face of limited testing and lengthy test turn-around times.^5,11^ Additionally, it has been established that patients with COVID-19 may be asymptomatic for some time, creating the potential for patients to escape established screening processes and testing.^2^ These issues present challenges for OHNS departments attempting to standardize risk mitigation strategies during the pandemic response. As testing with faster turn-around times becomes more readily available, there is opportunity for the development of preoperative screening and testing policies for OHNS procedures. For example, following the present study period of data collection (March 18–April 8, 2020), with the increasing availability of efficient COVID-19 tests, all 3 medical centers developed systemwide standardized protocols for universal preoperative COVID-19 testing of all scheduled essential cases.

Aerosol-protective PPE use during this time also reflects developing perioperative risk mitigation strategies during the immediate COVID-19 response period. We defined PPE as eye protection and an N95 mask. On March 23, 2020, the AAO-HNS recommended that otolaryngologists limit their practice to only urgent or emergent care, treat any patient with unknown COVID-19 status as COVID-19 positive, and have necessary PPE for all procedures.^23^ The AAO-HNS stated that, based on the experiences of OHNS departments during the SARS-1 pandemic in 2003, N95 masks are necessary for patients who are undergoing airway surgery and have suspected or confirmed COVID-19 positivity.^24^ While OHNS societal recommendations call for appropriate PPE for all staff during any potential AGP, independent of patient COVID-19 status,^11^ the operationalization of this recommendation was hindered in the earliest stages of the COVID-19 response period by a shortage of available supplies. Among cases for which PPE data were available at our institutions, aerosol-protective PPE was worn by surgeons in 28.6% of general adult cases, 90% of pediatric cases, and 15.5% of CCC cases. The low aerosol-protective PPE utilization numbers reflect significant nationwide concerns during the immediate COVID-19 response period regarding PPE availability resulting from the national shortage.^15,16^ Additionally, the establishment of recommendations regarding the use of aerosol-protective PPE selection is a multifactorial process. Differences among institutional N95 utilization reflect many contributing variables, including availability of aerosol-protective PPE, procedure type, hospital policy, and surgeon preference. The present study findings reaffirm the need for standardization of perioperative risk management protocols, including aerosol-protective PPE usage, among OHNS providers during the pandemic response period. Immediately following the present study period of data collection (March 18–April 8, 2020), with the increasing availability of PPE, OHNS departments across all 3 centers developed standardized protocols for universal use of aerosol-protective PPE for all AGPs, regardless of the patient’s COVID-19 status. Data from this phase of the COVID-19 response are currently being analyzed and will be reported in a separate publication.

Definitive airway management data during this time demonstrate a delay in the development of aerosolization risk-minimization strategies in the immediate COVID-19 response period. Across all 3 sites, intubation was performed in the majority of cases. One general adult case (7.1%) involved jet ventilation, and 23 pediatric cases (16.2%) involved spontaneous ventilation (**Table 1**). Existing literature on COVID-19 transmission has described intubation as a procedure with one of the highest risks of viral transmission.^11^ Jet ventilation airway management also poses a high risk of viral transmission, as the patients’ airways are unobstructed without an endotracheal tube and aerosolized particles have fewer barriers to their spread in a positive pressure–ventilated open airway. In the present study, 50 pediatric cases (35.2%) involved bag mask ventilation. This form of airway management also exposes surgeon and staff to aerosolized particles through the intermittent covering and uncovering of patient’s upper aerodigestive tract throughout a procedure. Guidelines for preferred airway management for OHNS recommend closing circuits, minimizing bag mask ventilation, and avoiding awake intubation.^5^ Additionally, researchers discourage THRIVE, jet ventilation, or positive-pressure ventilation without a cuffed tracheal tube.^5^ Such guidelines on best practices for airway management must be made abundantly clear to OHNS and anesthesia departments early on during pandemic response efforts.

Several barriers exist to the operationalization of standardized protocols for aerosol-protective PPE and COVID-19 testing in the setting of OHNS. From the experience of OHNS departments at pediatric and adult academic medical centers, we identified availability of rapid COVID-19 testing and adequate aerosol-protective PPE to be significant limitations to operationalizing society recommendations. Large tertiary academic medical centers specifically face a host of challenges to the rapid integration of standardized testing and equipment requirements. The larger the care center, the more that levels of leadership and policy changes are necessary for the operationalization of new initiatives. The integration of preoperative COVID-19 testing into perioperative workflows is therefore a complex issue with multiple contributing limiting factors. Standardized protocols recommended by OHNS societies should reflect the various stages of pandemic response. For example, as fast COVID-19 testing and PPE become more readily available through enhanced production and sterilization processes, preoperative COVID-19 testing and aerosol-protective PPE for all otolaryngologic procedures should become standards of practice. The present academic medical centers adopted these practices starting April 9, 2020, when testing and PPE were more readily available. Preoperative COVID-19 testing became a universal requirement for all OHNS cases, and recycling policies with check-in/check-out rules for N95 masks were instituted. The present study represents an opportunity for international OHNS leadership to better define barriers to operationalization of pandemic response measures and to improve the design of emergency preparedness and response planning.

There are several limitations to the present study. PPE information that was not readily available in the electronic medical record was collected retrospectively by asking attending surgeons to recall their PPE usage for each case. Additionally, detailed intubation information across all 3 sites was not available to researchers (rapid sequence intubation, preoxygenation status, etc). The academic centers studied herein also present unique geographic considerations. The centers are located directly across from the Batelle N95 sterilization processes. We are aware that ready access to these resources has afforded our institutions opportunities.

The present study represents an analysis of OHNS experiences during the COVID-19 pandemic across pediatric and adult academic medical centers. In the present study, we examine perioperative management in the COVID-19 pandemic response immediately following the national mandate to suspend all elective cases. The pandemic response led to decreased case volume and a shift in the nature of surgery performed, from elective to nonelective/urgent cases. In the field of OHNS, the majority of essential surgical cases remained high-risk AGPs. During this initial response period, preoperative COVID-19 testing was performed and PPE worn by surgeons for a limited number of cases. These practices reflect a misalignment between OHNS society recommendations and the reality of hospital operations during a time of international COVID-19 testing and PPE shortages. OHNS departments responded by creating standardized protocols for universal COVID-19 testing and PPE usage. The findings of the present study highlight the need to create gold standards of preoperative screening for COVID-19 status, perioperative PPE usage, and airway management for OHNS procedures during pandemic response periods. Additionally, further definition is needed for essential versus nonessential cases as well as staffing requirements in the field of OHNS as the country transitions toward national reopening.
